# Early anti-inflammatory intervention ameliorates axial disease in the proteoglycan-induced spondylitis mouse model of ankylosing spondylitis

**DOI:** 10.1186/s12891-017-1600-7

**Published:** 2017-05-30

**Authors:** Hsu-Wen Tseng, Tibor T. Glant, Matthew A. Brown, Tony J. Kenna, Gethin P. Thomas, Allison R. Pettit

**Affiliations:** 1 The University of Queensland Diamantina Institute, Translational Research Institute, 37 Kent Street, Woolloongabba, QLD 4102 Australia; 2 Mater Research Institute-The University of Queensland, Faculty of Medicine, Translational Research Institute, 37 Kent St, Woolloongabba, QLD 4102 Australia; 3 Section of Molecular Medicine, Department of Orthopedic Surgery, Rush University Medical Center, 1735 W. Harrison Str., Cohn Research Building, Chicago, IL 60612 USA; 4 Institute of Health and Biomedical Innovation, Queensland University of Technology: Translational Research Institute, 37 Kent St, Woolloongabba, QLD 4102 Australia; 5 Charles Sturt University, Boorooma Street, Wagga Wagga, NSW 2678 Australia

**Keywords:** Ankylosing spondylitis, Spondyloarthropathy, Osteoproliferation, Early intervention, Proteoglycan-induced spondylitis mouse model

## Abstract

**Background:**

Ankylosing spondylitis (AS) is characterised by immune-mediated arthritis and osteoproliferation, ultimately leading to joint ankylosis. Whether inflammation is necessary for osteoproliferation is controversial, fuelled by the unclear efficacy of anti-inflammatory treatments on radiographic progression. In proteoglycan-induced spondylitis (PGISp), a mouse model of AS, inflammation is the prerequisite for osteoproliferation as osteoproliferation was only observed following inflammation-driven intervertebral disc (IVD) destruction. We hypothesised that early intervention with a potent anti-inflammatory therapy would protect IVD integrity and consequently alter disease progression.

**Methods:**

PGISp mice received vehicle or a combination of etanercept (ETN) plus prednisolone (PRD) therapy for 2 or 6 weeks initiated at an early disease stage. Peripheral arthritis was scored longitudinally. Spinal disease was assessed using a semi-quantitative histological scoring regimen including inflammation, joint destruction and excessive tissue formation.

**Results:**

ETN + PRD therapy significantly delayed the onset of peripheral arthritis. IVD integrity was significantly protected when treatment was commenced in early disease. Six-weeks of treatment resulted in trends towards reductions in intervertebral joint damage and excessive tissue formation. IVD score distribution was dichotomized, likely reflecting the extent of axial disease at initiation of therapy. In the sub-group of mice with high IVD destruction scores, ETN + PRD treatment significantly reduced IVD destruction severity, inflammation and bone erosion and reduced cartilage damage and excessive tissue formation.

**Conclusions:**

Early intervention with anti-inflammatory treatment not only improved inflammatory symptoms but also ameliorated structural damage of spine in PGISp mice. This preclinical observation suggests that early anti-inflammatory intervention may slow radiographic progression in AS patients.

**Electronic supplementary material:**

The online version of this article (doi:10.1186/s12891-017-1600-7) contains supplementary material, which is available to authorized users.

## Background

Ankylosing spondylitis (AS) is a debilitating spondyloarthropathy which predominantly affects the spine and pelvis. The disease initially presents as inflammation followed by osteoproliferation that can result in axial joint fusion and bone formation at inflamed entheses [1]. In many countries, first-line treatment recommendations involve the use of non-steroidal anti-inflammatory drugs (NSAIDs) and physiotherapy. Use of TNF-inhibitors is restricted to patients fulfilling the modified New York classification criteria for AS (i.e. presence of osteoproliferative changes in the sacroiliac joints required) [2] and who have failed NSAID therapy [3]. Both treatment approaches are effective at relieving inflammatory symptoms and suppressing objective measures of joint inflammation. However, it is not clear whether anti-inflammatory treatments retard progression of syndesmophyte formation, indicative of disease-associated osteoproliferation.

Recent reports have suggested that anti-TNF treatment can retard late stage disease [4, 5]. However, delayed diagnosis [6] and/or delayed treatment delivery [4], which frequently occur in AS, are associated with higher radiographic progression rates. Additionally, presence of syndesmophytes at anti-TNF treatment commencement is prognostic of radiographic progression [7] indicating that anti-TNF treatment may be less effective if structural damage is established. Hence, a “window of opportunity” [8] might exist early in AS during which optimal long-term benefit will be gained from initiating TNF-inhibitors or alternative anti-inflammatory therapies.

There is conflicting evidence regarding osteoproliferation dependence on inflammatory mechanism in AS. Anti-TNF treatment in the human leukocyte antigen-B27/human β2-microglobulin (HLA-B27/hβ2m) transgenic rat model decreased inflammatory symptoms but failed to inhibit activation of signalling pathways responsible for chondroproliferation [9]. Similarly, induction of osteophyte formation in the human TNF transgenic mouse arthritis model required the additional hit of Dickkopf-1 blockade [10]. The most compelling data comes from human magnetic resonance imaging (MRI) studies suggesting that whilst syndesmophytes are more likely to develop at vertebral corners with prior evidence of inflammatory or fatty lesions, many syndesmophytes occur without evidence of such changes [11]. However, the insufficient sensitivity of MRI to low grade inflammatory changes that may be the precursor of syndesmophyte formation, or the possibility that inflammation at vertebral corners is episodic in nature, limits the specificity confidence of this technical approach [12]. No superior imaging modalities are available and human histopathological studies are limited by the difficulties associated with accessing involved joints. Therefore, mouse models must be utilized to investigate the relationship between inflammation and osteoproliferation.

The proteoglycan-induced spondylitis (PGISp), a mouse model of AS, is accompanied with peripheral arthritis allowing a visual diagnosis of early inflammatory events [13]. An advantage of this mouse model is that both inflammation and osteoproliferation are evident in the spine [14]. Slow progression of structural changes and high heterogeneity between individual animals and between joints within the same individual, while experimentally challenging, are similar to disease progression and characteristics observed in AS patients [15]. Using this model, we recently reported that the axial disease initiated as a intervertebral joint associated inflammatory response leading to intervertebral disc (IVD) destruction. We postulated that IVD destruction subsequently increased axial mechanical stress, joint damage and ultimately excessive tissue formation, as an aberrant repair response [16]. Consequently, suppressing inflammation prior to irreparable IVD damage might be an effective therapeutic approach for preventing or ameliorating progression of AS [16].

The objective of this study was to interrogate whether early and aggressive anti-inflammatory treatment is effective at preventing inflammation and whether this ultimately prevented/reduced progression to osteoproliferation. To achieve this we used an aggressive combination anti-inflammatory therapy that was initiated in the early, predominantly inflammatory phase of disease in the PGISp mice AS model [16]. The combination anti-inflammatory therapy was supra-clinical dosing of etanercept (ENT, a soluble TNF decoy receptor) plus prednisolone (PRD, a potent glucocorticoid). Supra-clinical doses were utilised to achieve robust and broad spectrum immune-suppression. Impacts of this combination treatment over short- and long-term interventions were assessed using clinical scoring and semi-quantitative histopathological approaches.

## Methods

### Animals

All experimental procedures were approved by The University of Queensland animal ethics committee and adhered to by the Australian code for the care and use of animals for scientific purposes. In order to achieve 100% disease penetrance and robust severity, disease was induced as described previously [14] in 12-week-old IL4-deficient female BALB/c mice by intraperitoneal injection of partially purified cartilage extract (equivalent with 100 μg core protein of human cartilage proteoglycan (PG)) emulsified with 2 mg dimethyldioctadecylammonium bromide (DDA, Sigma-Aldrich, St. Louis, MO) in a total volume of 200 ml phosphate-buffered saline (PBS, pH 7.4) at day 0, and then boosted with 150 ml emulsion on weeks 3 and 6.

**Fig. 1:**
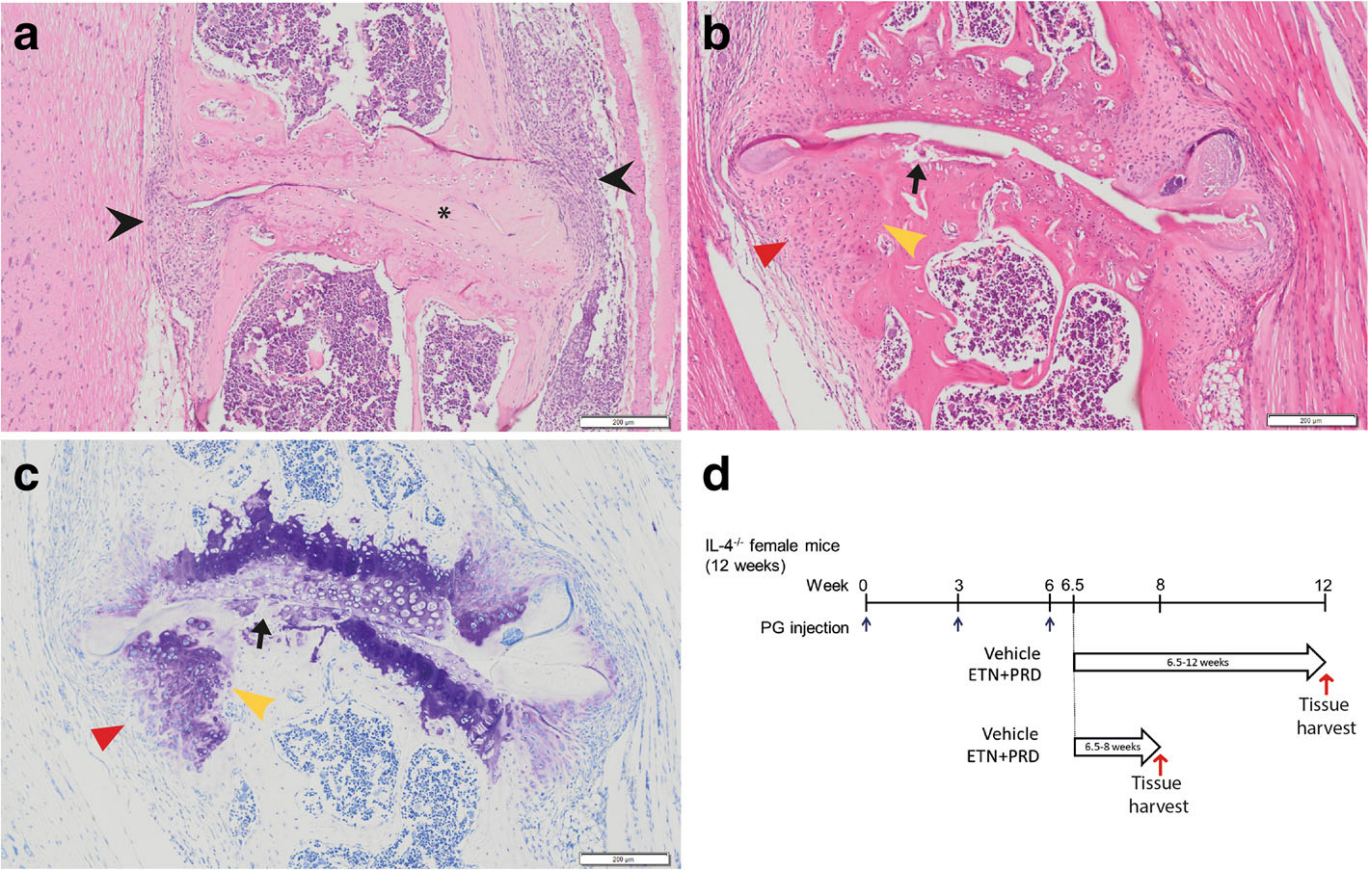
Axial disease progression and treatment regimen. Archetypical features of PGISp axial disease. **a** A representative image of an inflamed intervertebral disc (IVD) in which inflammatory cells (*black arrow head*) accumulate around the vertebral joint and invade the IVD space. Part of IVD has been destroyed, but there is still residual IVD tissue evident (*asterisk*). **b** H&E and **c** toluidine blue stained sections showing a representative example of a severely affected vertebral joint in which the IVD is destroyed with clear evidence of cartilage end plate (*black arrows*) and bone destruction (*yellow arrow heads*) plus evidence of osteoproliferation (*red arrow heads*). *Scale bars*: 200 μm. **d** Schematic representation of PGISp induction and anti-inflammatory treatment regimen. PGISp mice were treated with vehicle or ETN + PRD started 3 days after the third PG injection (“week 6.5”) and continued either until the end of week 8 (short-term) or week 12 (long-term) post-priming. Tissues were harvested at the end of each treatment period. Blue arrows indicate the initiation of treatment. Red arrows indicate tissue harvests. Open *arrows* indicate the treatment period

PGISp mice were treated with vehicle or ETN plus PRD (ETN + PRD). Mice were distributed randomly to treatment groups at first PG priming injection. ETN (provided by Pfizer, NY, USA) was administered at 10 mg/kg by subcutaneous injection 3 times a week. PRD was administered at 1.5 mg/kg/day by a slow-release pellet (Innovative Research of America, Sarasota, FL, USA) implanted subcutaneously (Fig. 1a). Vehicle groups were treated with PBS by subcutaneous injection with or without placebo pellets. All treatments started 6.5 weeks post the initial PG priming injection, so that the immune response was primed and re-activated in the absence of any anti-inflammatory treatment. ETN + PRD treatments were continued for 10 days (short-term, 6.5–8 weeks) or ~6 weeks (long-term, 6.5–12-weeks) (Fig. 1a). Peripheral inflammatory symptoms were scored ranging from 0 to 3 based on the degree of redness and swelling three times weekly. Cumulative arthritis scores of the four paws are presented as per previously [17]. Mice were euthanized at the end of each of the assigned treatment regimens and tissues collected for histological analysis.

### Histology and semi-quantitative score

Dissected thoracic to lumbar spines were decalcified, processed and sectioned (4 μm) in the sagittal plane. Sections were processed for standard haematoxylin and eosin (H&E) and toluidine blue staining as described previously [16].

A semi-quantitative histological scoring criterion based on H&E stain and toluidine stain was used to assess axial disease progression [16]. Briefly, the score for an intervertebral joint was produced by averaging the scores of ventral (anterior) and dorsal (posterior) aspects. An overall average score for each animal was then generated by averaging the scores for all assessable intervertebral joints in each sample (range 6 to 24 intervertebral joints scored in each sample (median = 13)). *Inflammatory score*: 0, no evident inflammatory infiltrate; 1, inflammation present at periphery of the IVD; 2, inflammatory pannus smaller than 50% IVD area; 3, inflammatory pannus invading more than 50% IVD area. *IVD destruction score*: 0, no evident disc destruction; 1, less than 50% disc destruction; 2, more than 50% disc destruction; 3, complete disc destruction. *Bone erosion score*: 0, no erosion; 1, one or a few small areas of resorption in original vertebral bone; 2, numerous areas of obvious focal resorption in original vertebral bone or several areas of severe destruction. *Cartilage damage score*: 0, no evident cartilage damage; 1, some loss of endplate cartilage and/or growth plate cartilage; 2, severe loss of endplate cartilage and some growth plate cartilage damage. 3, severe loss of both endplate and growth plate cartilages. *Excessive tissue formation score*: 0, no evident mesenchymal cells or chondrocytes around IVD; 1, mesenchymal cell invasion/expansion; 2, fibrocartilage formation smaller than 50% of the original disc area; 3. fibrocartilage formation larger than 50% of the original disc area. Intervertebral joints that scored one or greater in any of the criteria described above were defined as affected joints. PGISp mice with at least one involved inter-vertebral joint were considered as affected mice. The incidence of axial disease was the percent of affected mice within the group. The axial disease penetrance was defined as the percent of affected intervertebral joints within an individual mouse. Based on expert biostatistical advice from QFAB Bioinformatics, further subgrouping of mice into low and high disease activity was based on median IVD destruction score (vehicle: 1.05 and ETN + PRD: 0.66).

### Statistics

Mann-Whitney tests were used to determine statistically significant differences in univariate analyses. The relationship between histological features was determined by Spearman correlations. These statistical analyses were performed using PRISM 6 (GraphPad Software, La Jolla, CA). The statistical significant differences between the proportions of outcomes between treatment groups were determined using one-tailed Z-tests (http://epitools.ausvet.com.au/content.php?page=home). *P* values less than 0.05 were considered significant in the present study.

## Results

### Anti-inflammatory intervention dampened systemic inflammation in the PGISp model

We designed and implemented an early and intensive anti-inflammatory therapeutic approach in the PGISp model. Our intention was to initiate treatment post disease initiation but prior to irreversible IVD destruction [16]. In the absence of useful serum markers of axial disease progression (Additional file 1: Figure S1), we selected the treatment commencement point based on our previously reported histology evidence showing that 60 and 100% of PGISp mice exhibited axial inflammation (Fig. 1a) by weeks 6 and 8 post initial priming, respectively. Clear progression of IVD destruction (Fig. 1a) was seen in more than 50% of mice by 8 weeks post-priming with peak IVD destruction severity reached by 10 weeks with 100% of mice affected [16]. Extensive osteoproliferation (Fig. 1b and c) was observed at the affected intervertebral joints initiating 8 weeks post initial priming [16]. Accordingly, the treatment regimen in the present study was initiated 6.5 weeks post-priming to achieve a “therapeutic” delivery prior to development of irreversible IVD changes. Impacts of both short- and long-term therapies were examined with mice harvested either 2 or 6 weeks post-treatment initiation (Fig. 1d). The proportion of mice with no disease or peripheral and/or axial disease within each therapy arm was determined (Table 1). Clinical scores of peripheral joints were used to longitudinally assess systemic disease activity. Axial disease was assessed by histopathology with detection of one or more of the scored histopathological features being deemed as presence of axial disease. Eight weeks after PG priming, 80% of vehicle treated mice had evidence of peripheral and/or axial disease; in contrast, only 14% of mice in the ETN + PRD treated group had both. All mice developed evidence of disease 12 weeks post-priming irrespective of treatment. In the vehicle-treated group, 100% exhibited both peripheral and axial disease. In contrast, in ETN + PRD-treated mice only 62.5% had peripheral plus axial disease, indicating greater variance in systemic disease after early anti-inflammatory therapy. Taken together, these observations indicate that early treatment with ETN + PRD had robust short-term efficacy.

**Table 1:** Incidence of peripheral and axial disease

Treatment (treatment period)	Vehicle (6.5–8)	ETN + PRD (6.5–8)	Vehicle (6.5–12)	ETN + PRD (6.5–12)
Both peripheral and axial disease	80%	14.3% (*p* = 0.01)	100.0%	62.5% (*p* = 0.035)
Peripheral disease only	0	14.3%	0.0	25%
Axial disease only	20%	42.9%	0.0	12.5%
Presence of axial disease	100%	57.1% (*p* = 0.02)	100.0%	75% (*p* = 0.0015)
Incidence of peripheral disease	80%	28.6%	100%	87.5%
Peripheral disease incidence at treatment commencement	40%	28.6%	57.1%	62.5%
Total number	5	7	7	8

The statistical significance of data was analysed by one-tailed Z-tests compared with vehicle at corresponding time points

*Abbreviations*: *ETN* etanercept, *PRD* prednisolone

### Early ENT-PRD intervention transiently suppressed severity of peripheral arthritis

**Fig. 2:**
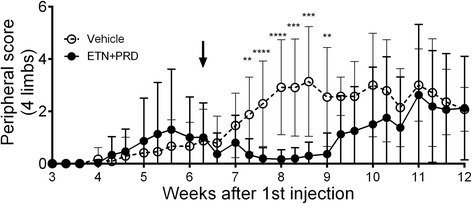
Early intervention delays and suppresses peripheral disease progression. Peripheral arthritis progression of mice receiving vehicle (open circle, *N* = 12) and ETN + PRD (filled circle, *N* = 15) in both treatment arms. The line up to the week 8 point includes mice from both the short-term and long-term treatment groups, as these animals are directly comparable up to this point. The line from week 9 to 12 includes only long-term treatment group mice. Cumulative arthritis score (inflammation of all 4 limbs of all animals are shown (mean ± SD). Animal numbers per group are listed in Table 1

The onset of peripheral arthritis varied between individual mice, with the earliest onset observed 4 weeks post priming (Fig. 2) and no significant difference was observed in peripheral disease incidence at the time of therapy initiation between any of the treatment groups (Table 1). In vehicle-treated mice (Fig. 2, dashed line), the peripheral arthritis score increased from 4 weeks post-priming followed by an accelerated progression after the third PG injection and then “levelled off” until the end of experimental period. In ETN + PRD-treated mice (Fig. 2, solid line) the initial acute arthritis (between weeks 4 and 6.5) was suppressed immediately after the first treatment, and remained negligible for 2–3 weeks. Within this period (from the beginning of ETN + PRD until week 9), there was a significant difference in peripheral paw inflammation between the vehicle-injected and ETN + PRD-treated groups (Fig. 2, *p* < 0.05). Hence, the ETN + PRD treatment regimen delayed and suppressed the peripheral disease development.

### Early ENT + PRD intervention suppressed axial inflammation and ameliorated both destructive and osteoproliferative disease outcomes

**Fig. 3:**
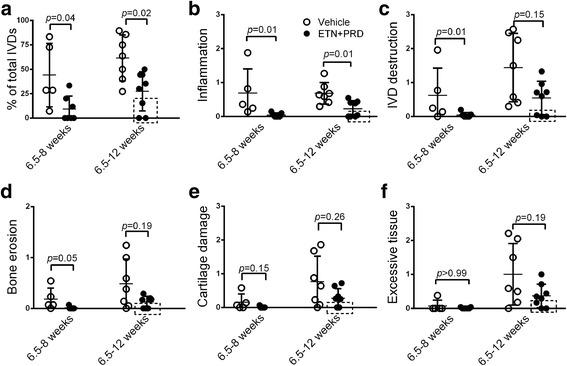
Early anti-inflammatory intervention ameliorates axial disease progression. **a** Penetrance (percent of affected inter-vertebral joints within all inter-vertebral joints scored in the same spine) and the histological scores of **b** inflammation, **c** IVD destruction, **d** bone erosion, **e** cartilage damage, and **f** excessive tissue formation. Vehicle (open circle. 6.5–8 weeks, *N* = 5; 6.5–12 weeks, *N* = 7) and ETN + PRD (filled circle, 6.5–8 weeks, *N* = 7; 6.5–12 weeks, *N* = 8). Each symbol represents one mouse in the group and the results are presented as mean ± SD. Symbols in dashed boxes indicate mice with minimal axial disease. The statistical significance of data was analysed by Mann-Whitney analysis

ETN + PRD treatment impacted the incidence, penetrance, severity and progression of the axial disease. Specific features of axial disease activity in each animal were assessed using semi-quantitative histological scoring of spine sections. Due to disease severity being variable between intervertebral joints within individual animals (Additional file 2: Figure S2), global impact of treatments on the specific disease features was assessed by generating an average histopathological feature score for each spine (Additional file 2: Figure S2). ETN + PRD treatment significantly reduced the penetrance of axial disease (percent of affected intervertebral joints within an individual animal) in both short-term and long-term treatment arms (Fig. 3a).

When compared with vehicle controls, ETN + PRD treatment significantly suppressed axial inflammation in both treatment arms (Fig. 3b) supporting the effectiveness of the ETN + PRD regimen on spondylitis. IVD destruction (Fig. 3c), bone erosion (Fig. 3d), and cartilage damage (Fig. 3e) were evident in both short- and long-term vehicle control groups (Fig. 3f). Within the short-term ETN + PRD treatment, scores for IVD destruction and bone erosion were significantly lower than controls with a similar trend toward reduction in cartilage damage and excessive tissue formation (Fig. 3).

In the long-term treatment arm, scores for disease features in the vehicle treated group were non-parametric and exhibited a wide degree of variation (Fig. 3). When data points for the different histopathological features were paired for individual animals, it was clear that a subset (3 of 8 mice, Fig. 3, data points highlighted by the dashed boxes) of ETN + PRD treated mice had minimal or undetectable axial disease as indicated by no or little evidence of disc destruction, bone erosion, cartilage damage or excessive tissue formation (Fig. 3, boxed area). All 3 of these mice had detectable peripheral disease confirming successful disease induction.

**Fig. 4:**
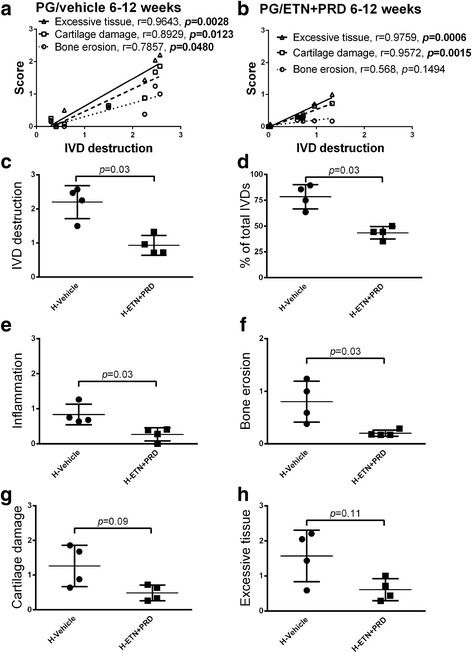
Early intervention reduced axial disease in more severely affected mice. **a** IVD destruction is strongly correlated with bone erosion (circle), cartilage damage (square) and excessive tissue formation (triangle) in long-term vehicle-treated PGISp mice (*N* = 7)**.** The positive correlation is also present in the ETN + PRD group, with the exception of bone erosion (*N* = 8) **b**. PGISp mice in vehicle group were sub-grouped into low disc destruction and high disc destruction (H-Vehicle, filled circle, *N* = 4) in relation to the median score (1.50). ETN + PRD-treated mice were split into low disc destruction and high disc destruction (H-ETN + PRD, filled square, *N* = 4) in relation to the median score (0.66): Semi-quantitative histological scores for **c** IVD destruction, **d** percentage of affected IVDs, **e** inflammation, **f** bone erosion **g** cartilage damage and **h** excessive tissue formation. Each symbol represents one mouse in the group and the results are presented as mean ± SD. The statistical significance of data was analysed by Mann-Whitney analysis

While inflammation is not a robust indicator of axial disease progression with a non-linear disease process, IVD destruction is an irreversible event, and therefore its presence is a constant mark of previous disease activity. In the long-term vehicle treatment arm, IVD destruction scores significantly and positively correlated with bone erosion, cartilage damage and excessive tissue formation scores (Fig. 4a). In ENT + PRD long-term treated mice the correlation of IVD destruction with cartilage damage and excessive tissue formation was upheld, but bone erosion scores no longer correlated (Fig. 4b). Interrogation of the IVD destruction data distribution in the long-term treatment arm supported dichotomization of this data set with mild and severe disease courses defined by low versus high IVD destruction. Consequently sub-groups were segregated based on the median IVD destruction score (vehicle: 1.05 and ETN + PRD: 0.66).

When comparing the low IVD destruction sub-groups, there was no significant difference between treatments across all histopathological criteria (not shown). When comparing high (H)-ETN + PRD with H-Vehicle, the mean IVD destruction score was significantly lowered by ETN + PRD (Fig. 4c). Furthermore, H-ETN + PRD had significantly lowered percent of affected IVD (Fig. 4d), inflammation (Fig. 4e) and bone erosion scores (Fig. 4f). Trends toward treatment-induced lowering of cartilage damage (Fig. 4g) and excessive tissue formation (Fig. 4h) were also noted. Overall, in this sub-group of mice that had a more severe clinical progression, early intervention with ETN + PRD treatment ameliorated axial disease severity.

## Discussion

The PGISp mouse model of AS was used to address whether early intervention of an aggressive anti-inflammatory drug regimen could alter broad aspects of the disease course, particularly retardation of irreversible joint changes. The study was also designed to inform on the dependence between early inflammatory processes and ongoing destructive changes in this AS model [16]. We observed delayed progression and reduced severity of peripheral disease in the first 2–3 weeks of ETN + PRD treatment. Histological analysis demonstrated that this transient systemic suppressive effect of anti-inflammatory treatment was associated with longer term significant reductions in axial inflammation, number of affected axial joints and vertebral joint destruction as well as declining trends in excessive tissue formation. Notably, the chosen anti-inflammatory treatment also clearly decreased disease activity compared to vehicle even in mice that exhibited a more severe disease course.

In the present study, early intervention with ETN + PRD clearly decreased bone erosion and weakened its association with IVD destruction. These observations provide further support that this destructive disease outcome is a direct result of the inflammatory process [16]. While IVD destruction was significantly ameliorated by the anti-inflammatory therapy it was not completely abated, and in joints with compromised IVD, progressive excessive tissue formation was observed. This provides support in favour of the hypothesis that IVD destruction-induced structural instability plays an important role in driving osteoproliferative outcomes. Increased intervertebral body mechanical forces as a consequence of IVD damage/loss may promote local bone formation through differentiation of osteoprogenitor cells as a consequence of elevated mechanosensing signals [18]. This is in agreement with clinical studies showing that presence of syndesmophytes when treatment commenced strongly predicted radiographic progression regardless of treatment [7].

The PGISp model recapitulates many key features of AS, including variability in disease onset and heterogeneity in disease severity both between and within individuals [15, 16]; while this variability makes it a good AS model, it confounded the current study outcomes by reducing statistical power. Peripheral disease is not an accurate predictor of axial disease and consequently cannot be used to reliably inform appropriate treatment initiation. In the absence of a sufficiently sensitive in vivo longitudinal imaging approach to detect spinal inflammation, therapy initiation could not be uniformly initiated at a defined and consistent threshold of axial disease development. Therefore treatment initiation was based on a fixed time post-priming that was selected based on our previously reported axial disease time course data [16]. Study outcomes were further limited by the fact that the length of study, which was dictated by anti-inflammatory therapy delivery limitations, did not extend to permit peak attainment of osteoproliferative tissue formation in the vehicle control group [16]. Consequently the current study was underpowered for robust assessment of some disease features. Nevertheless, the data provide clear evidence of beneficial impact of an early and aggressive anti-inflammatory intervention across a broad range of AS-like disease characteristics. Extended time course studies in larger cohorts will be required to definitively dissect the effects of early anti-inflammatory interventions on late-stage disease outcomes.

The results of the current study provide further support to the concept that inflammation and osteoproliferation in AS are dependent but sequential events. Accordingly the results advocate that early intervention with effective anti-inflammatory treatment in AS has a high potential to prevent both primary joint destruction and secondary osteoproliferative responses. However this study does not preclude usefulness of anti-inflammatory treatments in established disease. Specifically, aggressive anti-inflammatory therapy did ameliorate disease in mice that had a more progressed and severe disease course.

## Conclusion

This study highlights the importance of improving early diagnostic methods, particularly with respect to axial inflammation, in order to inform better clinical management of AS.

## Additional files

Additional file 1: Figure. S1. Serum IL-17 and CRP concentration does not changed during disease progression. Serum samples were collected from 6, 8, 10, 12, 16, 24 weeks post the first PG injection as reported previously [16] (A) Serum IL-17 was measured using the IL-17A ELISA MAX™ Deluxe set (BioLegend) as per the manufacturer’s instructions. (B) Serum CRP concentrations were analysed using mouse CRP ELISA DuoSet kit (R&D system, DY1829, Minneapolis, Minn., USA) as per the manufacturer’s instructions. There was no significant difference between naïve and any time points post PG-priming. Data is presented as mean ± SD. The statistical significance was analysed by Kruskal-Wallis test followed by Dunn’s multiple comparisons test. (TIFF 54 kb)

Additional file 2: Figure. S2. Representative images of IVDs with more severe axial disease. Representative images of H&E stained sections containing a portion of each spine within the field of view. A representative mouse exhibiting more severe axial disease severity scores from each of the PGISp experimental groups is shown: (A) vehicle 6.5–8 weeks, (B) ETN + PRD 6.5–8 weeks, (C) vehicle 6.5–12 weeks (D) ETN + PRD 6.5–12 weeks. The fields of view exemplify the variability between intervertebral joints within individual spines including unaffected joints with preserved IVDs (open arrows). A and B) In the short-term treatment groups inflammatory features are still prominent as illustrated by presence of immune cell infiltrates (arrow heads) in both (A) vehicle and (B) ETN + PRD treated groups. In (A) inflammatory scores for each of the intervertebral joints denoted (a) through (d) were 2, 2.5, 1.5 and 0 respectively, with 4 of the 11 intervertebral joints that were scored for this sample shown (total spine score for inflammation in this sample was 0.82). In (B) inflammatory scores for intervertebral joints denoted (a) through (c) were 0, 0.5 and 1 respectively, with 3 of the 24 intervertebral joints scored for this sample shown (total spine score for inflammation in this sample was 0.15). C and D) In long-term treatment groups, IVD destruction and excessive tissue formation (black arrows) are evident in both vehicle (C) and ENT + PRD (D) treated animals. In (C) excessive tissue formation scores for intervertebral joints denoted (a) through (e) were 2, 1.5, 0, 2 and 2 respectively, with 5 of the 8 intervertebral joints scored for this sample shown (total spine score for excessive tissue formation in this sample was 1.44). In (D) excessive tissue formation scores for intervertebral joints denoted (a) through (f) were 1, 0, 0, 0, 0 and 2 respectively, with 6 of the 17 intervertebral joints scored in this sample shown (total spine score for excessive tissue formation in this sample was 0.29). Scale bar (A-B) 500 μm (C-D) 1 mm. (TIFF 25040 kb)

## Abbreviations

AS: Ankylosing spondylitis
DDA: Dimethyldioctadecylammonium bromide
ETN: Etanercept
H&E: Haematoxylin and Eosin
HLA-B27/hβ2m: Human leukocyte antigen-B27/human β2-microglobulin
IVD: Intervertebral disc
MRI: Magnetic resonance imaging
NSAIDs: Non-steroidal anti-inflammatory drugs
PGISp: Proteoglycan-induced spondylitis mouse model
PRD: Prednisolone
TNFα: Tumour necrosis factor alpha
