# Supplementary figures and images for: Early anti-inflammatory intervention ameliorates axial disease in the proteoglycan-induced spondylitis mouse model of ankylosing spondylitis
Source: BMC Musculoskelet Disord. 2017 May 30;18:228. doi: 10.1186/s12891-017-1600-7 (PMC5450150; doi:10.1186/s12891-017-1600-7)

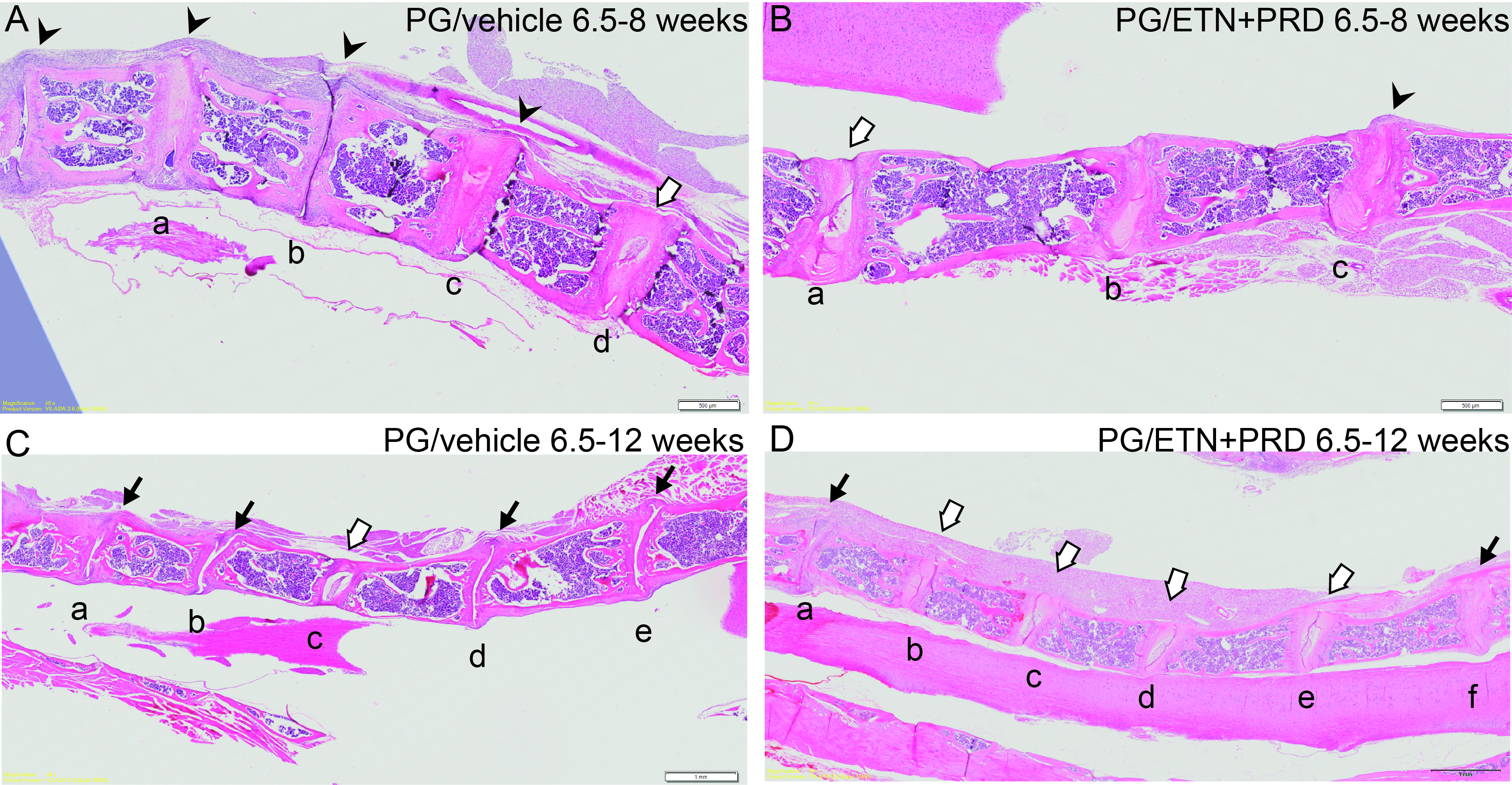

Supplement: Supplementary file 2 — Representative images of IVDs with more severe axial disease. Representative images of H&E stained sections containing a portion of each spine within the field of view. A representative mouse exhibiting more severe axial disease severity scores from each of the PGISp experimental groups is shown: (A) vehicle 6.5–8 weeks, (B) ETN + PRD 6.5–8 weeks, (C) vehicle 6.5–12 weeks (D) ETN + PRD 6.5–12 weeks. The fields of view exemplify the variability between intervertebral joints within individual spines including unaffected joints with preserved IVDs (open arrows). A and B) In the short-term treatment groups inflammatory features are still prominent as illustrated by presence of immune cell infiltrates (arrow heads) in both (A) vehicle and (B) ETN + PRD treated groups. In (A) inflammatory scores for each of the intervertebral joints denoted (a) through (d) were 2, 2.5, 1.5 and 0 respectively, with 4 of the 11 intervertebral joints that were scored for this sample shown (total spine score for inflammation in this sample was 0.82). In (B) inflammatory scores for intervertebral joints denoted (a) through (c) were 0, 0.5 and 1 respectively, with 3 of the 24 intervertebral joints scored for this sample shown (total spine score for inflammation in this sample was 0.15). C and D) In long-term treatment groups, IVD destruction and excessive tissue formation (black arrows) are evident in both vehicle (C) and ENT + PRD (D) treated animals. In (C) excessive tissue formation scores for intervertebral joints denoted (a) through (e) were 2, 1.5, 0, 2 and 2 respectively, with 5 of the 8 intervertebral joints scored for this sample shown (total spine score for excessive tissue formation in this sample was 1.44). In (D) excessive tissue formation scores for intervertebral joints denoted (a) through (f) were 1, 0, 0, 0, 0 and 2 respectively, with 6 of the 17 intervertebral joints scored in this sample shown (total spine score for excessive tissue formation in this sample was 0.29). Scale b [file 12891_2017_1600_MOESM2_ESM.tif]
